# *Ex vivo* evaluation of the accuracy of four electronic foraminal locators

**DOI:** 10.4317/jced.61003

**Published:** 2024-01-01

**Authors:** Ilana-Thais-de Freitas Lima, Ana-Letícia-Linhares-de Sousa Paula, Reuton-dos Santos Palheta-Filho, Amanda-Brito Santos, Nathalia-de Aguiar Freitas, George-Táccio-de Miranda Candeiro

**Affiliations:** 1 MSC, Department of Dentistry, Unichristus, Fortaleza, Ceará, Brazil; 2 DDS, Department of Dentistry, Unichristus, Fortaleza, Ceará, Brazil; 3 Department of Dentistry, Unichristus, Fortaleza, Ceará, Brazil; 4 PhD, Department of Dentistry, Unichristus, Fortaleza, Ceará, Brazil

## Abstract

**Background:**

To evaluate the accuracy of four different Electronic Foraminal Locators (EFLs): Root ZX II (J. Morita, Tokyo, Japan), Romiapex A15 (Romidan, Kiryat-Ono, Israel), FinePex (Schuster, Porto Alegre, Brazil), and VDW Gold (VDW, Munich, Germany), in determining root canal length.

**Material and Methods:**

Twenty-seven human single-rooted teeth had their crowns sectioned at the cementoenamel junction, and the actual length of the tooth was obtained by visualizing with an operative microscope a #15 file placed adjacent to the apical foramen. The teeth were instrumented with R25 and R40 files, and at the end of each instrumentation, measurements of the lengths of the root canals were made with #25 and #40 files. The data were statistically analyzed by ANOVA and chi-square tests and were considered significant when *P*<0.05. All devices showed a tendency to underestimate measurements by 0.25 mm diameter.

**Results:**

The mean accuracy of the Root ZX II appliance was statistically lower than the other EFLs (*P*<0.001). For the 0.40 mm diameter, the mean accuracy of the Romiapex A15 appliance was statistically higher than the other LEFs (*P*<0.001). However, when the diameter was 0.40 mm, only the Romiapex A15 device tended to overmeasure. Regarding the acceptable limits of variation, it was observed that the devices showed similar efficiency in determining odontometry (*P*>0.05), both with diameters of 0.25 mm and 0.40 mm.

**Conclusions:**

It was concluded that the devices presented similar and adequate efficacy when observing the limits of acceptable measurements. It was observed that increasing the apical diameter did not influence the accuracy of EFLs in determining root canal lengths.

** Key words:**Endodontics, working length, electronic foraminal locators, endodontic treatment.

## Introduction

During endodontic treatment, correctly determining root canal length is critical and essential for success (1). Historically, the working length (WL) was determined by radiographic means, but given several limitations of these methods, Electronic Foraminal Locators (EFLs) was developed to determine the WL more accurately and reliably (2) This device operates based on the electrical impedance between the oral mucosa and periodontal ligament (3). It is one of the most reliable methods described in the literature, with an accuracy greater than 90% under ideal conditions (4).

Currently, the market has several EFL devices of different brands and models. The Root ZX II (J. Morita, Tokyo, Japan) is one of the most widely used EFL in Europe, Asia, and the United States (5) and is considered the gold standard. It calculates the ratio between impedances measured at two frequencies (8KHz and 0.4 KHz), which justifies its high accuracy and sensitivity (6). It became a pioneer as it presented superior advantages over its predecessors (7).

The RomiApex A15 is a device that operates differently from most EFLs by detecting the energy of the signal rather than its amplitude. First, it measures the CT by calculating the mean values of the square root of the impedance at different frequencies (0.5 and 8.0 kHz). Then, it compares the results obtained with the reference values stored in its memory and thus determines the file’s position inside the canal (8).

Over the years, EFLs integrated into mechanized systems, such as the VDW Gold engine, have also emerged. This system can be conFigured for measurements in manual mode, just like standard EFLs, or for controlled mode in conjunction with mechanical instrumentation (9). Although the Root ZX II has undergone exhaustive research proving its effectiveness, few studies are found in the literature on the accuracy of the RomiApex A15 and VDW Gold motor devices. Recently, a localizer called FinePex has been launched in the market, with no studies evaluating its effectiveness so far.

Therefore, this study aimed to evaluate the precision of four EFLs using different operating mechanisms, Root ZX (J. Morita, Tokyo, Japan), Romiapex A15 (Romidan, Kiryat-Ono, Israel), Finepex (Schuster, Porto Alegre, Brazil) and VDW Gold motor (VDW, Munich, Germany), in two moments, after instrumentation with files from the Reciproc R25 system and after instrumentation with R40.

## Material and Methods

Initially, the present study was submitted and approved by the Research Ethics Committee of the Christus University Center under protocol number 3.099.081 (ANNEX 1).

Twenty-seven human single-rooted teeth, extracted for reasons unrelated to the research, were collected for this study. The teeth had fully formed apices, with apical diameters smaller than 0.25 mm, without resorption, calcification, dilaceration, and previous endodontic treatment. The teeth remained immersed in saline solution until the moment of use.

The crowns of the teeth were then cut using a diamond disk (KG Sorensen, Cotia, Brazil) with a chuck attached to a straight piece of low rotation, standardizing the root length at 17 mm. After standardizing the specimens, coronary access was performed with diamond tips No. 1013 and No. 3081 (KG Sorensen, Cotia, Brazil) on teeth with full crowns. The canals were explored with hand files K #10 (Dentsply Maillefer, Ballaigues, Switzerland). A clinical microscope (Alliance, São Carlos, Brazil) with 10x magnification, a K#15 file, and a digital caliper (Mitutoyo, Suzano, Brazil) were used to determine the actual tooth length (CRD) and obtain the foraminal patency, which was used as a reference for the other measurements.

The cervical and middle thirds were prepared with CP Drill (Helse Dental Technology, São Paulo, Brazil). The specimens were coupled in an acrylic device and embedded in a medium (Carbopol Gel, 0.9% NaCl, and 2% KCl - FarmaVie Farmácia de Manipulação, Fortaleza, Brazil).

The chemical-surgical preparation (PQC) was performed in the CRD (Reading 1) with an R25 file coupled to the VDW Gold motor (VDW, Munich, Germany), and a saline solution was used. The excess irrigating solution was removed with EndoFlex endodontic suction (Maquira, Maringá, Paraná). Reading 2 was performed employing the LEFs Root ZX II (J. Morita, Tokyo, Japan), Romiapex A15 (Romidan, Kiryat-Ono, Israel), FinePex (Schuster, Porto Alegre, Brazil), and VDW Gold (VDW, Munich, Germany) using the K#25 file. The measurements were made in triplicate to obtain the average between the measurements. The measurements were made with the file inserted slowly into the root canal until the “OVER” signal was obtained, and then the instrument was retracted until the “APEX” or “0.0” signal was reached. After five seconds of stability, the silicone slider of the file was positioned, and the file was measured with a digital caliper. The measurements were recorded in Microsoft Excel® spreadsheets.

Next, the root canals were prepared with the R40 instrument attached to the VDW Gold motor (VDW, Munich, Germany), and saline solution was used. A third reading was performed similarly to the previous reading, with a K#40 hand file employed for measurement.

After measurements were tabulated and spreadsheets of the Microsft Excel program, the quantitative data were subjected to the Kolmogorov-Smirnov normality test, expressed as mean and standard deviation, and analyzed by ANOVA test followed by the Bonferroni post-test. Then, the measurements were categorized based on the clinical relevance of measuring the size of the dental unit between - 0.50 and + 0.50, between -1.00 and + 1.00, and between -1.50 and + 1.50. Data were expressed as absolute and percentage frequency and analyzed by the chi-square test.

## Results

**Figure 1:**
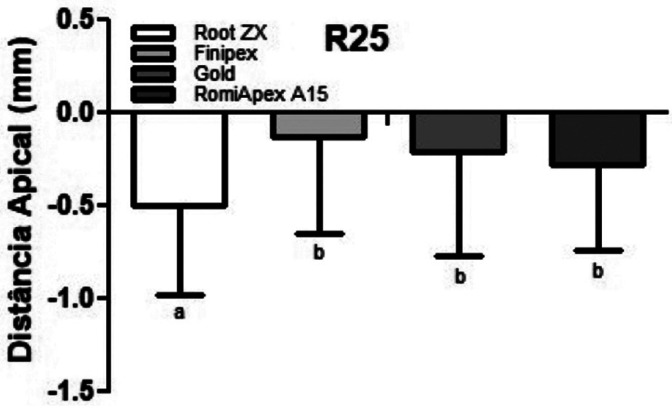
Means and standard deviation of the apical distance obtained by four different systems after instrumentation with the R25 file.

Figure 1 presents the mean and standard deviation after PQC with files from the Reciproc R25 system. All the appliances tended to under-measurements with the 0.25 mm diameter (Table 1, Fig. 1). The mean accuracy of the Root ZX II appliance was statistically lower than the other LEFs (*P*<0.001). Table 2 shows the values of the measurements observed in each measurement, divided by distances from the apical foramen with 0.25 mm diameter.

**Table 1:**
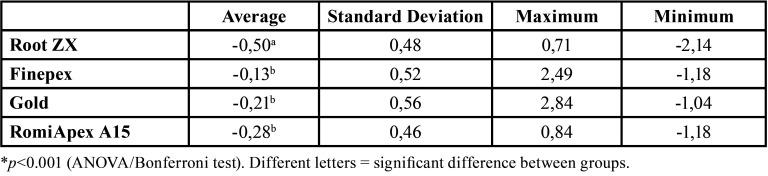
Means and standard deviation of the apical distance obtained by four different systems after instrumentation with the R25 file.

**Table 2:**
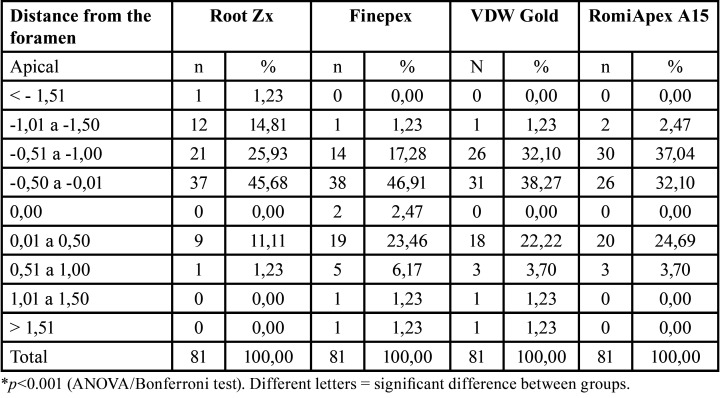
Analysis by distances of the apical foramen (measurements with 25.02).

**Figure 2:**
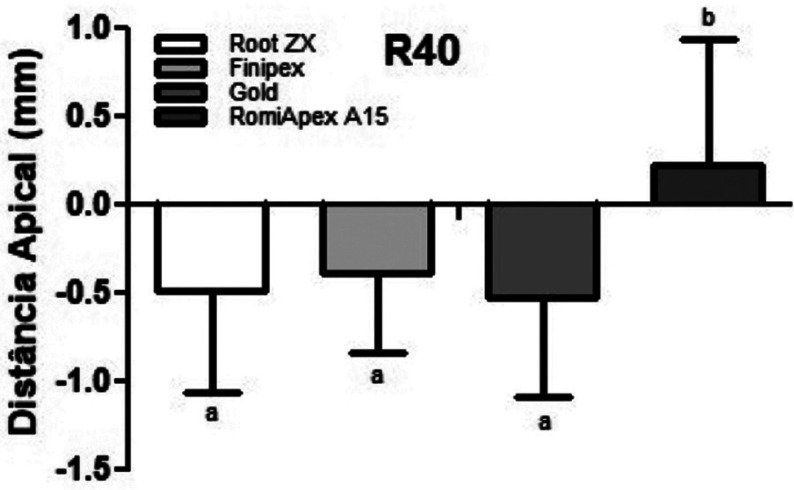
Means and standard deviation of the apical distance obtained by four different systems after instrumentation with the R40 file.

At 0.40 mm diameter, the mean accuracy of the Romiapex A15 device was statistically higher than the other EFLs (*P* < 0.001). However, when the diameter used was 0.40 mm, only the Romiapex A15 device tended to overmeasure (Table 3, Fig. 2). Table 4 shows the values of the measurements observed in each measurement, divided by distances from the apical foramen with 0.40 mm diameter.

**Table 3:**
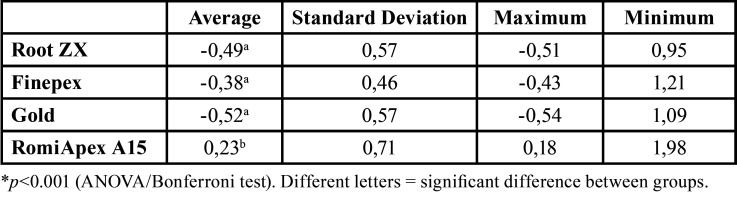
Means and standard deviation of the apical distance obtained by four different systems after instrumentation with the R40 file.

**Table 4:**
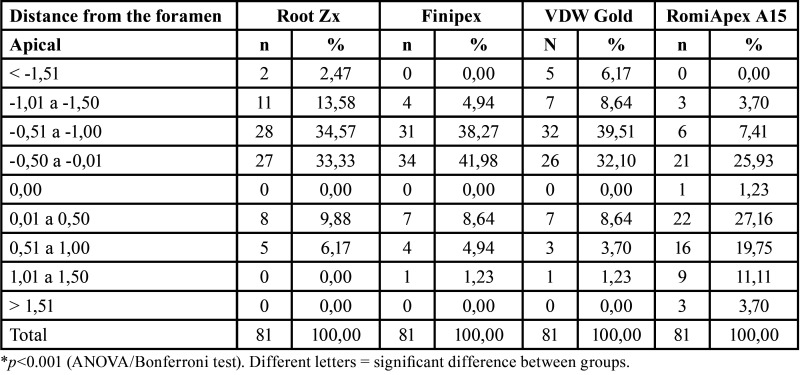
Analysis by distances of the apical foramen (measurements with 40.02).

Regarding the accepTable limits of variation, it was observed that the devices showed similar efficiencies in determining odontometry (*P*>0.05), both with diameters of 0.25 mm and 0.40 mm (Table 5). In all appliances, the efficiency of the measurements was directly proportional to the increase in the accepTable variation limit, with efficiencies ranging from 40.74% to 100%, depending on the limit to be considered. The only device that significantly changed the mean readings as the diameter of the foramen increased was the Romiapex A15 (*P*<0.001).

**Table 5:**
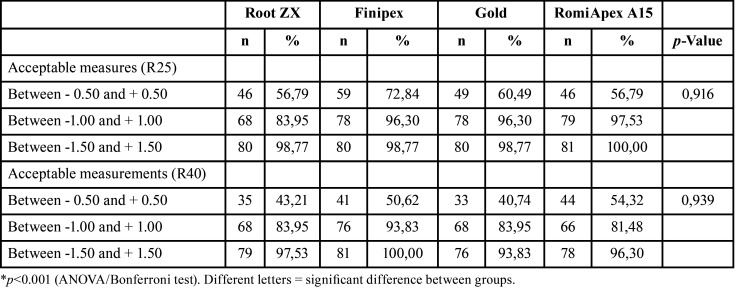
Absolute and percentage frequency of the apical distance levels obtained by four different systems after instrumentation with R25 and R40 files.

## Discussion

In today’s endodontics, using EFLs is indispensable since correctly determining the working length is related to successful endodontic treatment (10,11). Using EFLs decreases the working time and the radiation dose on the patient (3). Another advantage of EFLs is their applicability under challenging situations, such as in patients with vomiting and with difficulties in visualizing the root apices due to some anatomical structure or other factors, such as an unerupted tooth or a metal fixation plate.

The methodology used in the present study was based on several previous works (12-15). Single-rooted teeth are commonly used in similar studies for standardization and decreased bias (15). Most studies to evaluate EFLs are ex vivo studies, as they have advantages in their conduct over *in vivo* studies due to the ease of maintaining strict control over experimental conditions. In addition, a greater number of root canals and appliances can be tested. When comparing the accuracy of the Root ZX II *in vivo* and *in vitro* groups, observed no statistically significant differences between these groups (16).

Several factors, such as the isolation quality, the previous cervical preparation, the absence of metal restorations and the presence of moisture in the pulp chamber, and the adaptation of the instrument to the canal walls in the apical portion can influence the accuracy of EFLs. Other factors still need to be more questionable and require further research. In the present study, pre-widening of the cervical and middle thirds was performed to favor electronic determinations of EFLs, as suggested in previous works (17,14,15).

Although Ebrahim *et al*. (12) found lower efficacy of EFLs with larger foramen, in the present study, the increase in the apical diameter of instrumentation did not significantly influence the efficacy of the devices. Only the Romiapex A15 appliance showed higher frequencies of over measurements, however, within the same accuracy limit.

Comparing the accuracy of EFLs is challenging, as several variables can influence root canal measurements.18 Studies show that the Root ZX II is a reliable and accurate device under adverse conditions, such as steep root canal curvature (19,20). Duran-Sindreu *et al*. (16), when comparing the accuracy of the Root ZX II in an *in vivo* and *in vitro* group, observed no statistically significant differences between these groups. Morgental *et al*. (17) observed that pre-widening the cervical third is advantageous in the accuracy of LEFs; the best results of these appliances are obtained after cervical preparation (14). However, Maia-Filho *et al*. (21), when comparing the accuracy of Root ZX II and RomiApex A15, observed that these two appliances showed similar accuracy, considering RomiApex A15 a reliable option for CT determination. Considering a tolerance of ± 0.5mm of the actual lengths, Silva & Alves (22) considered that the Root ZX II produced more accepTable measurements than the Root ZX Mini and RomiApex A15. In the present work, it was observed that when the apical diameter was referring to a #25 instrument, the accuracy of Root ZX II was statistically lower than the other devices. However, when the diameter corresponded to a #40 file, the Romiapex A15 accuracy was statistically higher than the other appliances. However, within the accepTable limits of ± 0.5 mm, ± 1.0 mm, and ± 1.5 mm, the devices did not present differences in reading effectiveness.

The electronic measurement was determined when the tip of the instrument surpassed the apical foramen, marked in the devices as an “OVER” signal, and retracted until it reached 0.0 signal, which means the position of the apical foramen. Oliveira *et al*. (15) found higher percentages of accuracy (83% and 93%) when the apical foramen was used as an anatomical landmark, regardless of the operating mechanism of the appliance. Vasconcelos *et al*. (13), when evaluating the accuracy of five different EFLs, observed that all devices provided accepTable measurements in the 0.0 position, a fact also observed in the present study.

After instrumentation and before odontometry, foraminal patency was performed to clear the apical foramen. According to Vasconcelos *et al*. (23), the absence of foraminal patency affects the accuracy of EFLs. This suggests that dentin plugs hinder the passage of current through the root canal, preventing the electric current from reaching the apical foramen and thus significantly interfering with the accuracy of EFLs.

This differs from the results of Guise *et al*. (24), who compared the accuracy of three different EFLs and observed that Root ZX II showed greater accuracy in determining root length. In this study, Root ZX II showed statistically lower accuracy than the other EFLs; however, it showed greater stability in the two apical diameters.

Current EFLs feature high accuracy rates and are considered safe and reliable (18). However, each manufacturer has a different operating mechanism. Vasconcelos *et al*. (13) found differences in devices that operate with similar mechanisms.

To date, no studies have evaluated the accuracy of the FinePex device. In the present study, this appliance provided adequate results to be safely used in the clinic. This appliance showed a tendency for sub-measurements and was little influenced by the increase in apical diameter. The Finepex appliance still showed acceptable measurement frequencies with ± 0.5 mm and ± 1.0 mm.

It was observed in this study that all devices showed a tendency to sub-measurements with a diameter of 0.25mm. Such factor can be explained by Vasconcelos *et al*. (14), who concluded that the root canal length presents a significant reduction after PQC, making it necessary to perform electronic measurements before the obturation of the root canals once the instrumentation has been completed.

Miletic *et al*. (8) reported that the RomiApex A15 would not be reliable as the sole means of determining working length. Nevertheless, in the present study, this device tended to overmeasure and have a higher accuracy at 0.40 mm diameter. When the apical diameter was 0.25 mm, RomiApex A15 showed higher accuracy than Root ZX II and was similar to Finepex and VDW Gold. However, no significant difference in efficacy was observed between the devices tested, and RomiApex A15 is suitable for clinical use.

Only one study was found evaluating the accuracy of the VDW Gold in manual mode. Ali *et al*. (9) observed no significant differences in the CT measurements of this device in manual mode and the auto-reverse function; this device presented measurements within the margin of error clinically acceptable, corroborating the present study’s data.

Martins *et al*. (25) considered little scientific evidence available on using EFLs but concluded that EFLs reduce patient exposure to radiation and are the best method for determining working length. Miletic *et al*. (2011), when comparing the reproducibility of EFLs (RomiApex A15, Dentaport ZX, and Raypex 5), concluded that EFLs are unreliable as the only method for CT determination under clinical conditions. Thus, research should be conducted so that possible variations can be verified and the use of EFLs can be optimized.

## Conclusions

From the methodology employed in the present work and given the results presented, we can conclude that:

• The devices showed similar efficacy when the limits of acceptable measurements were observed.

• All devices tended to under-measure when 0.25 mm diameters were used.

• Only the Romiapex A15 device tended to over-measure when 0.40 mm diameters were used.

• Increasing the apical diameter did not influence the efficacy or the accuracy of the appliances tested.
